# The Impact of Concussions on Neuromuscular Control and Anterior Cruciate Ligament Injury Risk in Female Soccer Players: Mechanisms and Prevention—A Narrative Review

**DOI:** 10.3390/jcm14093199

**Published:** 2025-05-05

**Authors:** Georgios Kakavas, Nikolaos Malliaropoulos, George Skarpas, Florian Forelli

**Affiliations:** 1 Fysiotek Spine & Sports Lab, 17562 Athens, Greece; georgios.kakavas@gmail.com; 2 ErgoMech-Lab, Department of Physical Education and Sport Science, University of Thessaly, 38221 Volos, Greece; 3 Thessaloniki Sports Medicine Clinic, 54622 Thessaloniki, Greece; contact@sportsmed.gr; 4 Sports Clinic, Rheumatology Department, Barts Health NHS Trust, London E1 1BB, UK; 5 Department of Orthopaedics, Hellenic Open University, 10677 Athens, Greece; skarpasg@gmail.com; 6 Haute-Ecole Arc Santé, HES-SO University of Applied Sciences and Arts Western Switzerland, 2800 Delémont, Switzerland; 7 Orthopaedic Surgery Department, Clinic of Domont, Ramsay Healthcare, OrthoLab, 95330 Domont, France; 8 Société Française des Masseurs—Kinésithérapeutes du Sport Lab, 93380 Pierrefite sur Seine, France

**Keywords:** concussion, anterior cruciate ligament injury prevention, neuromuscular impairment, proprioception, return-to-play, female soccer players

## Abstract

**Background/Objectives:** Soccer players, particularly females, exhibit an increased risk of both concussions and Anterior Cruciate Ligament (ACL) injuries. Emerging evidence suggests that neurcognitive deficits following concussions may impair neuromuscular control, increasing ACL injury susceptibility. This study aims to explore the interplay between concussions, neuromuscular deficits, and ACL injury risk, while proposing targeted prevention strategies. **Methods:** A comprehensive review of current literature was conducted to analyze the biomechanical and neurophysiological impact of concussions on ACL injury risk. Key areas investigated include the effect of sub-concussive impacts on proprioception, reaction time, and postural stability, as well as sex-based differences in injury susceptibility. **Results:** Findings indicate that post-concussion neuromuscular impairments—such as altered proprioception, delayed reaction times, and compromised joint stability—heighten ACL injury risk. Female athletes, due to biomechanical and hormonal factors, are particularly vulnerable. Preventive measures, including neuromuscular training, cervical spine strengthening, and optimized return-to-play protocols, are essential to mitigate these risks. **Conclusions:** Longitudinal research is needed to further elucidate the connection between head trauma and ACL injuries. Implementing evidence-based interventions and policy changes, such as modifying heading exposure in youth athletes, may enhance player safety and reduce long-term injury burden in female soccer players.

## 1. Introduction

Soccer has undergone a remarkable surge in popularity over the past three decades, particularly among female athletes, with participation rates in high school and collegiate sports reaching unprecedented levels [1,2]. While the sport offers significant physical and psychosocial benefits, it also presents substantial injury risks, particularly for female players who demonstrate a higher incidence of both concussions and Anterior Cruciate Ligament (ACL) injuries compared to their male counterparts [3,4].

Concussions, including both symptomatic and sub-concussive impacts, are among the most prevalent injuries in soccer, with female players at an increased risk due to biomechanical and physiological differences such as lower neck strength, reduced head mass, and hormonal fluctuations that may affect neurocognitive function and recovery [5,6]. The effects of concussions extend beyond immediate neurological symptoms, potentially influencing motor control, balance, and neuromuscular coordination—factors that are critical in the prevention of lower extremity injuries [7,8]. Recent evidence suggests that even mild traumatic brain injuries can disrupt sensorimotor integration and postural stability, potentially predisposing athletes to subsequent musculoskeletal injuries, including ACL ruptures [9,10]. The persistence of neurocognitive deficits, even after symptom resolution, raises concerns regarding the adequacy of return-to-play protocols in mitigating secondary injury risk [11].

Female soccer players exhibit ACL injury rates up to six times higher than their male counterparts, a disparity often attributed to anatomical, neuromuscular, and biomechanical factors, such as increased knee valgus angles, lower hamstring-to-quadriceps strength ratios, and differences in neuromuscular activation patterns [12]. However, an emerging body of research proposes that prior concussions may exacerbate these risks by altering neuromuscular timing, proprioceptive control, and reaction speed, thereby increasing susceptibility to non-contact ACL injuries [8,11]. Sub-concussive impacts, such as repetitive heading of the ball, may contribute to cumulative neurophysiological changes that impair cognitive-motor function, further elevating the likelihood of lower extremity injuries [13,14,15]. These findings underscore the potential interplay between brain trauma and lower extremity injury mechanisms, emphasizing the need for an integrated approach in injury prevention.

Understanding the relationship between concussions and ACL injuries may provide useful insights for developing targeted prevention strategies. Given the potential for prolonged neuromuscular impairments following head trauma, incorporating neurocognitive assessments into injury risk evaluations may provide valuable insights into athletes’ readiness to return to play [16]. Additionally, neuromuscular training programs designed to enhance postural stability, proprioception, and dynamic joint stabilization may serve as a critical intervention to mitigate the cascading effects of concussions on ACL injury risk [17,18]. Furthermore, advancements in wearable sensor technologies and motion analysis may offer objective measures to monitor sub-clinical deficits in motor function, providing a more comprehensive assessment of injury susceptibility [14,15].

Given the emerging evidence linking concussions to musculoskeletal injury risk, particularly ACL injuries, further research into the underlying mechanisms appears warranted. This review aims to synthesize current literature on the relationship between concussion and ACL injury risk, highlighting potential pathways through which sub-concussive impacts may influence musculoskeletal vulnerability and proposing targeted interventions to mitigate these risks. Addressing these topics through interdisciplinary research and evidence-based practice may contribute to improving athlete safety and reducing sports-related injuries among female soccer players.

## 2. Methods

This narrative review was conducted with the aim of synthesizing existing literature on the relationship between concussions, neuromuscular impairments, and ACL injury risk in female soccer players, as well as to identify evidence-based prevention strategies. The review followed a structured, yet non-systematic approach, appropriate for exploring an emerging and multifactorial topic that spans across biomechanics, neuroscience, and sports medicine.

A comprehensive literature search was performed between December 2023 and February 2024 using three major databases: PubMed, Scopus, and Web of Science. Search terms included combinations of the following keywords: “concussion,” “ACL injury,” “neuromuscular control,” “sub-concussive impacts,” “proprioception,” “reaction time,” “female athletes,” “soccer” and “injury prevention.” Boolean operators (AND/OR) were applied to combine terms appropriately.

The inclusion criteria were as follows:

− Peer-reviewed articles published in English between 2000 and 2024
− Studies involving human subjects, with a focus on female soccer players or female athletes in similar high-risk sports
− Articles addressing neuromuscular consequences of concussion or sub-concussive impacts
− Research related to ACL injury mechanisms, risk factors, or prevention strategies
− Systematic reviews, narrative reviews, clinical trials, cohort studies, and relevant position statements or consensus papers

Studies focusing exclusively on male athletes, those not addressing the link between neurocognitive function and lower-extremity injury, or those with insufficient methodological detail were excluded.

No formal quality appraisal tool (e.g., PRISMA or GRADE) was applied due to the narrative nature of the review. However, studies were selected based on their scientific relevance, sample characteristics, design quality, and contribution to the overall conceptual framework. Reference lists of included studies were also screened for additional relevant sources.

Findings were then thematically organized into the following core areas:

- The biomechanical and neuromuscular consequences of concussion and sub-concussive impacts
- The influence of heading and fatigue on lower limb mechanics
- Sex-specific factors contributing to concussion and ACL injury risk
- Epidemiological trends across age groups and competitive levels
- Injury prevention and rehabilitation strategies
- Gaps in current research and future methodological recommendations

This structured yet flexible approach allowed for the integration of interdisciplinary findings and the identification of critical areas where further research is needed.

## 3. Results

### 3.1. Ball Heading Contributes to Increased Anterior Cruciate Ligament Injury Risk

The relationship between heading in soccer and ACL injuries is an evolving area of research, with growing evidence suggesting that repetitive sub-concussive impacts may contribute to neuromuscular deficits that increase lower extremity injury risk [8,14,15]. While heading does not typically result in symptomatic concussions, cumulative exposure to these impacts has been associated with changes in brain function, including altered postural stability, proprioception deficits, and delayed reaction times, which may predispose players to ACL injuries [9,13].

- **The Influence of Repetitive Heading on Neuromuscular Control and ACL Injury Risk**

Emerging evidence suggests that repetitive heading in soccer predominantly impairs lower extremity biomechanics through disruptions in cortical motor control and sensorimotor integration, rather than via direct mechanical transmission of force from the ball impact. Sub-concussive impacts, although often asymptomatic, have been shown to alter white matter microstructure, cortical excitability, and neurocognitive performance [19], which collectively compromise an athlete’s ability to maintain postural control, proprioceptive accuracy, and dynamic stability [6].

Neurocognitive deficits following repetitive head impacts manifest as delayed neuromuscular responses, impaired anticipatory postural adjustments, and decreased precision in limb positioning [20]. These sensorimotor impairments are critical in the context of high-demand athletic movements such as sudden decelerations, cutting maneuvers, and single-leg landings, where milliseconds of delay or minor deviations in joint control can markedly elevate ACL strain [21].

Research has shown that individuals with a history of concussion exhibit greater medial knee displacement, reduced hip and knee flexion angles during dynamic tasks, and asymmetrical gait patterns compared to non-concussed athletes [22]. These biomechanical aberrations reflect impaired cortical regulation of motor commands and sensorimotor feedback, highlighting a central nervous system-driven mechanism increasing ACL injury risk.

Furthermore, cumulative sub-concussive impacts can exacerbate neuromuscular fatigue, a condition linked to slower motor unit recruitment and reduced joint stiffness [23]. Neuromuscular fatigue further diminishes proprioceptive acuity and reactive postural control, compounding the deleterious effects of cortical dysfunction on lower limb biomechanics.

Importantly, these alterations in movement mechanics and neuromuscular control occur even when athletes are deemed clinically recovered based on symptom resolution alone, suggesting that current return-to-play assessments may not adequately capture lingering sensorimotor vulnerabilities [24].

In conclusion, the link between repetitive heading and ACL injury risk is best understood as a neurophysiological cascade, beginning with sub-clinical disruptions in cortical and white matter function, leading to impaired sensorimotor integration, altered neuromuscular control, and ultimately dysfunctional lower extremity biomechanics.

Recognizing and addressing these neuromotor deficits may help in developing effective prevention programs. Future interventions should combine neuromuscular training with cognitive-motor integration drills and objective movement screening to safely reintegrate players into competition.

- **The Neuromechanical Impact of Fatigue and Sub-Concussive Head Impacts on ACL Injury Risk**

In female soccer players, the risk of ACL injury is multifactorial, involving biomechanical, neuromuscular, and neurocognitive factors. Recently, attention has shifted to the cumulative effects of sub-concussive head impacts and neuromuscular fatigue as key contributors to injury vulnerability. While sub-concussive impacts, such as repetitive soccer heading, may not produce immediate clinical symptoms, evidence suggests they cause subtle, persistent sensorimotor and cognitive impairments [25]. In parallel, neuromuscular fatigue—resulting from high training loads and match play—deteriorates proprioception, reaction time, and motor coordination [26]. The interplay between these two mechanisms may critically undermine dynamic joint stability and movement quality, progressively heightening the risk for non-contact ACL injuries. Despite their plausible connection, the combined effects of fatigue and head trauma on injury mechanisms remain poorly integrated in current research. A deeper understanding of these interactions is crucial for developing effective, targeted prevention strategies tailored to the specific needs of female athletes.

- **Effects of Fatigue on Neuromuscular Control**

Neuromuscular fatigue exerts profound effects on both central and peripheral components of motor control, undermining joint stability and increasing injury susceptibility. Fatigue impairs the central nervous system’s ability to activate motor units rapidly and synchronously, leading to slower reaction times, diminished reflexes, and decreased muscle stiffness [27]. Peripheral fatigue, characterized by metabolic changes within the muscle fibers themselves, further compounds these deficits by delaying force production and altering movement patterns [26].

Biomechanically, fatigued athletes have been shown to land with greater vertical ground reaction forces, increased medial knee displacement, and decreased hip and knee flexion angles [28]. These compensatory movement strategies shift loads away from the musculature toward passive structures such as ligaments and joint capsules, particularly the ACL. Moreover, proprioceptive acuity declines under fatigue, impairing an athlete’s ability to detect joint position changes and execute appropriate protective reflexes during dynamic actions. These neuromuscular deficits cumulatively degrade an athlete’s capacity to respond effectively to unexpected perturbations or mechanical stresses, particularly during high-demand tasks like cutting, pivoting, and single-leg landings.

### 3.2. Interaction Between Sub-Concussive Impacts and Fatigue

While the independent effects of sub-concussive impacts and neuromuscular fatigue have been well documented, few studies have explored how these mechanisms may interact synergistically to elevate injury risk. Sub-concussive head impacts, even in the absence of diagnosed concussion, are associated with impaired postural control, slower cognitive processing speeds, and alterations in cortical motor planning [29]. These deficits can prime the neuromuscular system for dysfunction, which is further exacerbated when fatigue supervenes.

Fatigue amplifies the underlying sensorimotor impairments caused by repetitive heading, increasing sway, slowing neuromuscular responses, and impairing bilateral coordination [25]. In such conditions, athletes are more likely to adopt compensatory movement patterns such as stiff landings or excessive valgus collapse during deceleration, which directly increase ACL loading. Critically, the subtle sensorimotor alterations induced by sub-concussive impacts may not be detectable by standard sideline assessments, meaning athletes may unknowingly return to play while already compromised. This compounded vulnerability underlines the importance of considering both cognitive and physical fatigue together in injury prevention frameworks.

### 3.3. Specific Biomechanical Consequences

The neuromechanical consequences of cumulative sub-concussive trauma and fatigue manifest in several specific, high-risk biomechanical patterns. Notably, fatigued and sensorimotor-impaired athletes consistently demonstrate increased dynamic knee valgus during landing and cutting tasks, characterized by medial displacement of the knee joint relative to the hip and ankle [30]. Excessive valgus loading places abnormal tensile stress on the ACL, particularly during rapid decelerations and changes of direction.

Additionally, athletes in fatigued or sensorimotor-impaired states exhibit reduced hip and knee flexion angles upon landing, resulting in stiffer, less absorptive mechanics [31].This stiffer posture increases vertical forces transmitted through the knee joint, overwhelming ligamentous structures designed for tension rather than compression. Asymmetrical limb loading, increased trunk sway, and delayed protective muscle activation patterns have also been observed, further compounding ACL strain risk [29]. Together, these maladaptive movement strategies create an environment where the ACL is repeatedly exposed to mechanical stress beyond its physiological tolerance.

### 3.4. Clinical Implications and Prevention Strategies

Given the multifactorial nature of the risks introduced by neuromuscular fatigue and sub-concussive exposure, adapting injury prevention strategies could be beneficial. Neuromuscular training programs that emphasize dynamic balance, reactive agility, and cognitive-motor integration have demonstrated efficacy in improving sensorimotor control and reducing ACL injury rates [32,33,34]. Specific interventions targeting hip abductor strength, trunk stabilization, and proprioceptive responsiveness are critical to correcting faulty movement patterns exacerbated by fatigue [35].

Fatigue management protocols should also be incorporated into daily practices, including monitoring training loads, implementing structured rest periods, and utilizing rotation strategies during match play [26]. Wearable technologies such as inertial measurement units and real-time gait analysis systems offer new opportunities to detect early signs of neuromuscular fatigue and movement asymmetry [36].

Finally, return-to-play criteria following head impacts should be expanded to include neuromuscular testing—such as dynamic postural control tasks and reactive movement assessments—to ensure that athletes have regained sufficient sensorimotor function before exposure to high-risk athletic activities.

### 3.5. Limitations of Current Literature and Future Research Directions

Although increasing evidence supports the role of neuromuscular fatigue and sub-concussive trauma in ACL injury risk, critical gaps persist. Most studies examining these factors are cross-sectional, limiting causal inferences. Very few have adopted longitudinal designs tracking neuromechanical changes across competitive seasons, and even fewer have focused on female populations despite clear sex-specific vulnerabilities [37].

Future research should prioritize longitudinal, multimodal monitoring combining cognitive assessments, biomechanical measurements, and wearable sensor data to map the progression of neuromuscular deterioration. Investigations should also examine the effects of integrated interventions targeting both cognitive and neuromuscular components, with an emphasis on female-specific biomechanics and neurophysiology. Developing predictive models integrating sensorimotor deficits and external workload measures could enable earlier identification of high-risk athletes and the implementation of personalized injury prevention strategies.

While cumulative neuromuscular fatigue and repetitive sub-concussive impacts broadly compromise sensorimotor control, dynamic joint stability, and overall movement quality, it is equally important to recognize the specific neurocognitive deficits induced by head trauma. Concussions and repetitive head impacts can disrupt cortical motor planning, proprioceptive integration, and reaction times, all of which are critical for safe athletic performance. The following section will explore in greater detail how concussion-induced neurocognitive impairments contribute to ACL injury risk, particularly in female soccer players.

- **The Impact of Neurocognitive Deficits on ACL Injury Risk**

Concussions and sub-concussive impacts not only affect cognitive function but also have significant implications for neuromuscular control, proprioception, and reaction times. Deficits in these areas can dramatically increase the likelihood of ACL injuries, particularly in female soccer players who already exhibit biomechanical vulnerabilities.

### 3.6. Altered Proprioception and Joint Stability

Proprioception, the body’s ability to sense joint position and movement, is crucial for maintaining dynamic stability, particularly during high-risk activities such as landing, pivoting, and sudden changes in direction. Following a concussion, sensory integration between the vestibular, visual, and somatosensory systems may be impaired, leading to diminished proprioceptive awareness and reduced knee joint stability [8,9]. This deficit may cause athletes to adopt compensatory movement patterns that inadvertently place excessive stress on the ACL. For instance, research has demonstrated that concussed athletes exhibit greater knee valgus angles and reduced knee flexion during landing tasks, both of which are recognized as key risk factors for ACL injury [11,13].

### 3.7. Delayed Neuromuscular Responses and Reaction Time

Concussions disrupt sensorimotor processing, leading to delayed muscle activation patterns that can compromise postural control and coordination. The latency in neuromuscular response times may impair an athlete’s ability to properly stabilize the knee during rapid deceleration or unexpected perturbations [7,14]. This delayed response increases the risk of non-contact ACL injuries, particularly in situations where precise neuromuscular timing is essential, such as reacting to sudden changes in gameplay direction or avoiding an oncoming opponent.

Female athletes, due to hormonal and biomechanical differences, may be particularly susceptible to these impairments. Studies suggest that post-concussive neuromuscular deficits persist for extended periods in women, which could contribute to a prolonged window of increased injury risk [6,38]. Furthermore, these deficits may not always be detected through conventional concussion assessments, underscoring the need for specialized neuromuscular testing in post-concussion evaluations.

### 3.8. Post-Concussion Gait and Movement Pattern Changes

Emerging evidence suggests that concussed athletes exhibit alterations in gait mechanics and movement symmetry, which can further exacerbate ACL injury susceptibility. Post-concussion gait analysis has revealed increased medial-lateral sway, altered stride lengths, and asymmetrical loading patterns, all of which may contribute to improper knee mechanics during high-impact movements [17,18]. These findings reinforce the importance of integrating neuromuscular rehabilitation protocols alongside traditional cognitive assessments in concussion recovery.

### 3.9. Implications for Injury Prevention and Rehabilitation

To mitigate the impact of neurocognitive deficits on ACL injury risk, it is essential to implement targeted rehabilitation and injury prevention strategies. Proprioceptive retraining should be prioritized, incorporating balance exercises, unstable surface training, and visual-motor coordination drills to enhance joint position sense and neuromuscular feedback mechanisms [39]. Additionally, reaction time drills that emphasize agility, quick decision-making, and rapid directional changes can aid in restoring neuromuscular responsiveness post-concussion, reducing the risk of improper movement patterns [40]. Gait and biomechanical assessments should be integrated into post-concussion screenings to evaluate movement asymmetries and joint loading strategies, identifying compensatory mechanisms that could predispose athletes to lower extremity injuries [33]. Furthermore, gradual return-to-play protocols should be enforced, ensuring that athletes undergo progressive neuromuscular testing before resuming high-risk activities such as cutting, pivoting, and jumping [40]. Given the complex interplay between concussions, neuromuscular impairments, and ACL injury risk, future research should focus on longitudinal assessments of post-concussion movement deficits and their correlation with lower extremity injuries. By refining return-to-play criteria and integrating neuromuscular screening protocols, sports medicine professionals can improve injury prevention strategies and reduce the burden of ACL injuries among female soccer players.

- **Exploring Epidemiological Data Across Different Age Groups and Competition Levels**

Understanding the epidemiological trends of concussions and ACL injuries across different age groups and levels of competition is essential for refining prevention strategies. Injury risk factors may vary depending on developmental biomechanics, neuromuscular maturity, exposure to competitive environments, and training intensities.

### 3.10. Injury Incidence in Youth, Collegiate, and Professional Players

Studies indicate that ACL injury rates and concussions vary significantly between youth, collegiate, and professional female soccer players. Youth athletes (under 18) experience lower absolute ACL injury rates compared to collegiate and professional players, but this discrepancy may be due to differences in reporting mechanisms, gameplay intensity, and neuromuscular control [1,8]. However, in adolescents, there is a sharp increase in ACL injuries as players enter puberty, likely due to musculoskeletal changes, increased joint laxity, and neuromuscular imbalances [9,41].

At the collegiate level, injury incidence peaks, with female athletes displaying significantly higher ACL injury rates than their male counterparts [3]. The heightened risk is attributed to increased match frequency, higher physical demands, and exposure to more aggressive gameplay tactics. Professional female soccer players exhibit some of the highest ACL injury rates, often due to accumulated microtrauma, extended playing careers, and greater exposure to high-intensity competition [12].

### 3.11. Competition Level and Injury Susceptibility

The level of competition significantly influences injury patterns. High school and collegiate athletes have been found to sustain more non-contact ACL injuries, often during training or match situations involving rapid deceleration, pivoting, and poor landing mechanics. Professional athletes, however, tend to experience ACL injuries in both contact and non-contact scenarios, with cumulative fatigue and chronic joint stress playing a larger role in injury risk [17,18].

Concussion incidence follows a similar trend, with higher rates reported among collegiate and professional female players compared to youth athletes. Increased heading frequency, greater ball speed, and more intense physical challenges contribute to these elevated concussion rates at higher competition levels [11,14,15]. Furthermore, studies suggest that female athletes in elite competition take longer to recover from concussions, potentially due to higher cumulative exposure to head impacts over their playing careers [6].

### 3.12. Longitudinal Trends and Risk Factors

Tracking injuries over multiple seasons has revealed that repeated exposure to head impacts and neuromuscular deficits from prior injuries contribute to an increased likelihood of future ACL ruptures. Athletes with a history of concussion or lower extremity injuries are at significantly higher risk of sustaining ACL injuries, with studies reporting nearly a twofold increase in injury recurrence among previously concussed players [7]. The accumulation of sub-concussive impacts over multiple playing seasons may exacerbate neuromuscular deficits, further elevating injury susceptibility [13].

### 3.13. Implications for Injury Prevention and Future Research

To effectively reduce the prevalence of concussions and ACL injuries in female soccer players, targeted injury prevention strategies must be adapted to different age groups and competition levels. Age-specific training and injury mitigation strategies should be emphasized in youth development programs, incorporating neuromuscular training, proprioceptive drills, and biomechanical assessments to reduce ACL injury risk before athletes reach their peak vulnerability during adolescence and early adulthood [42]. Longitudinal player monitoring is essential for tracking injury patterns across multiple seasons, with the integration of wearable technology and motion analysis tools helping to refine early detection protocols for high-risk athletes [43]. At the collegiate and professional levels, competition-specific prevention protocols should be implemented to address the unique demands of high-intensity gameplay, ensuring that neuromuscular recovery programs mitigate the cumulative impact of fatigue and repetitive stress [8]. Given the increased susceptibility of female athletes to prolonged post-concussion symptoms, targeted concussion management should be customized based on age, level of competition, and neuromuscular recovery assessments, ensuring that players safely reintegrate into sport. Furthermore, future epidemiological research should focus on the long-term impact of repeated sub-concussive trauma on neuromuscular function and ACL injury risk, particularly in elite female athletes. By expanding epidemiological analysis and refining injury prevention strategies tailored to different demographics, sports medicine professionals can significantly reduce the burden of concussions and ACL injuries, ultimately enhancing player safety and career longevity.

- **The Need for More Longitudinal Studies: Identifying Research Gaps and Future Methodologies**

Despite the growing body of research on the relationship between concussions, sub-concussive impacts, and ACL injuries, significant gaps remain in our understanding of the long-term effects of repeated head trauma on neuromuscular function. Current studies often rely on cross-sectional or short-term retrospective analyses, which fail to capture the cumulative impact of repetitive head impacts and neuromuscular deficits over time. Longitudinal studies are essential to track injury patterns, assess recovery trajectories, and refine prevention strategies.

### 3.14. Current Limitations in Research

Establishing a clear causal relationship between concussions and ACL injuries remains a significant challenge due to several limitations in current research. One major issue is the short-term focus of many studies, which typically analyze injury incidence over one or two seasons. This timeframe is insufficient to capture long-term neuromuscular decline and the progressive impact of cumulative head trauma on ACL injury risk [44]. Additionally, the lack of standardized measures in concussion diagnosis, ACL injury assessment, and neuromuscular testing protocols hinders the comparability of findings across studies, making it difficult to draw definitive conclusions. Another major barrier is the underreporting of concussions, particularly among youth and collegiate athletes, who may not recognize or disclose mild or sub-concussive impacts. This results in incomplete datasets and an underestimation of the cumulative effects of repetitive head trauma on neuromuscular function [3]. Lastly, limited female-specific research remains a critical gap, as most studies are based on male populations, despite female soccer players exhibiting higher rates of both concussions and ACL injuries. The lack of sex-specific data makes it challenging to develop targeted injury prevention and rehabilitation strategies for female athletes. Addressing these research limitations through standardized protocols, longer-term studies, improved concussion reporting, and sex-specific investigations is essential to advancing injury prevention in female soccer players.

### 3.15. Proposed Methodologies for Future Longitudinal Research

To overcome current research limitations and improve our understanding of the relationship between concussions and ACL injuries, future studies should adopt more comprehensive methodologies. Large-scale cohort studies should be implemented to track athletes over multiple years across different age groups and competition levels, enabling the identification of long-term trends in concussion history, neuromuscular performance, and ACL injury incidence. Additionally, the integration of wearable technology can enhance data collection by utilizing accelerometers and gyroscopic sensors to measure heading impact forces, gait alterations, and neuromuscular function in real time. Continuous monitoring of head acceleration events will provide valuable insights into cumulative exposure to sub-concussive impacts and their potential consequences [45,46].

Furthermore, advanced motion analysis and biomechanical assessments should be employed, incorporating 3D motion capture and force plate analysis to evaluate neuromuscular responses before and after concussions. These tools can help detect compensatory movement patterns that may increase ACL injury risk [47]. Complementary to this, neurocognitive and sensorimotor testing should involve repeated baseline and post-injury assessments to track reaction time, balance, and proprioception. Investigating whether prolonged neurocognitive deficits correlate with altered neuromuscular control will be crucial in refining injury prevention strategies [47].

Lastly, multi-center collaborations and data sharing will be essential to improving research validity and applicability. Establishing a global registry of concussion and ACL injury data can increase sample sizes, strengthen statistical power, and ensure more reliable findings. Standardizing injury classification criteria and assessment methods will further enhance comparability across studies, ultimately contributing to more effective and evidence-based prevention strategies in female soccer players [48].

### 3.16. Implications for Injury Prevention and Rehabilitation

The implementation of longitudinal studies would provide sports medicine professionals with critical insights into the long-term effects of concussions and neuromuscular impairments on ACL injury risk. By tracking athletes over extended periods, researchers can develop more precise return-to-play protocols, ensuring that players have fully regained neuromuscular control before re-entering competition [46]. This would help reduce the likelihood of premature return and secondary injuries. Additionally, longitudinal studies would allow for the identification of early neuromuscular biomarkers for injury susceptibility, enabling the development of individualized training programs that address specific movement deficiencies and post-concussion impairments [46,47].

Furthermore, optimizing injury prevention programs based on an athlete’s concussion history and biomechanical weaknesses would help mitigate the risk of ACL injuries through targeted interventions [33,49]. These studies could also inform policy changes in soccer regulations, such as implementing restrictions on heading exposure in younger athletes to minimize cumulative sub-concussive impacts, or refining ACL prevention programs to address the unique vulnerabilities of female players.

By addressing these gaps through robust longitudinal research methodologies, sports medicine professionals will be able to establish clearer connections between head trauma, neuromuscular dysfunction, and ACL injuries. This knowledge will ultimately contribute to reducing injury incidence and improving player safety across all levels of soccer, from youth development programs to elite professional competition.

- **Sex Differences Significantly Influence Concussion Incidence and Recovery**

Sex differences in concussion incidence, symptomatology, and recovery have been widely recognized in sports medicine research. Female athletes, particularly those involved in contact sports such as soccer, exhibit higher rates of concussion than their male counterparts, along with longer recovery times and greater symptom severity [3,4]. Understanding these sex-specific disparities is crucial for improving concussion management and injury prevention strategies.

- **Physiological and Anatomical Differences**

One of the primary factors contributing to sex differences in concussion risk is the variation in cervical spine and musculature between male and female athletes. Women typically have smaller head mass and reduced neck strength compared to men, which may result in greater head acceleration upon impact, thereby increasing susceptibility to concussions [5,6]. Studies have shown that neck strength is inversely related to concussion risk, with weaker neck musculature leading to increased head displacement and higher force transmission to the brain during impact [50].

Moreover, sex-related differences in brain structure and cerebral blood flow may influence concussion susceptibility and recovery. Female athletes tend to exhibit higher levels of cerebral perfusion, which could contribute to increased vulnerability to metabolic disturbances following brain trauma [6]. Additionally, differences in white matter integrity and axonal connectivity between sexes may result in varying neurocognitive responses to concussive injuries [51].

- **Hormonal Influence and Concussion Recovery**

Hormonal fluctuations, particularly those related to the menstrual cycle, have been proposed as another contributing factor to sex differences in concussion outcomes. Estrogen and progesterone, which regulate various neuroprotective and neuroinflammatory processes, may modulate the brain’s response to injury [38]. Some studies suggest that female athletes who sustain concussions during the luteal phase of their menstrual cycle—when progesterone levels are elevated—experience prolonged symptoms and delayed recovery compared to those injured during other phases [52].

Additionally, variations in hormonal regulation may influence neurotransmitter activity and cerebral metabolism following concussions. While research in this area remains inconclusive, understanding the hormonal impact on concussion susceptibility could inform more personalized injury management strategies for female athletes [38].

- **Neurocognitive and Symptomatic Differences**

Female athletes not only report a higher incidence of concussions but also exhibit more severe and prolonged symptoms compared to males. Commonly reported symptoms in women include greater headaches, dizziness, visual disturbances, and cognitive impairment [3,53]. Furthermore, female athletes tend to perform worse on visual memory tasks following concussions and experience greater declines in reaction time, suggesting sex-based disparities in neurocognitive function post-injury [54].

These differences may be attributed to sex-specific variations in neuroanatomy, including differences in corpus callosum thickness and neural connectivity patterns [6]. Additionally, sex hormones such as estrogen have been implicated in synaptic plasticity and neuroinflammation, which may influence post-concussion recovery trajectories [5].

- **Implications for Concussion Management and Prevention**

Given the distinct physiological, hormonal, and neurocognitive responses to concussions in female athletes, tailored approaches to concussion management may be more effective. Strategies that emphasize neck strengthening programs, visual tracking exercises, and individualized return-to-play protocols could help mitigate sex-specific risks associated with concussion [10,17]. Additionally, increased awareness and education on concussion reporting behaviors in female athletes may enhance early diagnosis and treatment adherence.

Future research should focus on refining sex-specific concussion assessment tools and exploring the interplay between hormonal cycles, neurocognitive outcomes, and long-term brain health in female athletes. By addressing these factors, sports medicine professionals can develop more effective interventions to protect female athletes from the short- and long-term consequences of concussive injuries.

## 4. Discussion

The evidence presented in this review highlights a multifaceted relationship between concussions, particularly sub-concussive impacts from heading, and ACL injuries in female soccer players. While the direct causal link between heading and ACL injuries remains under investigation, the cumulative neurological and biomechanical effects of repetitive impacts provide compelling evidence of a heightened injury risk. The integration of findings on sex-based concussion differences further emphasizes the unique vulnerabilities female athletes face, necessitating tailored prevention and management strategies.

- **Interplay Between Concussion, Neuromuscular Deficits, and ACL Injury Risk**

One of the most significant findings from recent research is that concussions impair neuromuscular control, proprioception, and postural stability—key elements in injury prevention [8,9,41]. These impairments persist beyond symptom resolution, leading to prolonged deficits in reaction time, joint stabilization, and dynamic movement control. This prolonged neuromuscular dysfunction is particularly concerning in soccer, where players frequently engage in high-risk movements such as rapid deceleration, pivoting, and jumping. If an athlete’s neurocognitive and motor control systems remain impaired following a concussion, they may be less able to properly execute these movements, thereby increasing ACL injury susceptibility [11,16].

Sub-concussive impacts, particularly those experienced during heading, may contribute to neuromuscular deficits over time. Studies suggest that repeated exposure to low-impact head trauma results in cumulative alterations in sensorimotor processing, which can degrade postural control and reaction time [13,14]. This suggests that even without symptomatic concussions, players who frequently head the ball could develop subtle motor impairments potentially increasing ACL injury risk.

- **Sex-Specific Factors Amplifying Injury Susceptibility**

The elevated incidence of concussions and ACL injuries in female soccer players compared to males highlights the role of sex-specific physiological and biomechanical factors. Women typically exhibit greater knee valgus angles, lower hamstring-to-quadriceps strength ratios, and different neuromuscular activation patterns, all of which increase ACL injury risk [12]. Additionally, female athletes have lower neck strength and smaller head mass, which can lead to higher head acceleration upon impact, thereby increasing concussion susceptibility [5,6].

Hormonal influences may further exacerbate these risks. Fluctuations in estrogen and progesterone levels throughout the menstrual cycle have been linked to changes in ligament laxity, neuromuscular control, and cognitive function [38]. Some studies suggest that female athletes who sustain concussions during the luteal phase of their cycle may experience prolonged symptoms and slower neuromuscular recovery, increasing their vulnerability to secondary injuries such as ACL ruptures [52].

The combination of prolonged post-concussive neuromuscular deficits, sex-specific biomechanics, and hormonal fluctuations creates a unique set of risk factors for female athletes. Rehabilitation programs and injury prevention strategies should account for these interrelated elements to effectively reduce ACL injuries following concussions.

- **Implications for Prevention and Future Research**

To address the elevated risk of ACL injuries in female soccer players, particularly in the context of concussions and sub-concussive impacts, a comprehensive approach must be implemented:

### 4.1. Implementing Neuromuscular Training Programs

Neuromuscular training programs should be a fundamental component of injury prevention strategies for female soccer players, given their effectiveness in reducing ACL injury risk and mitigating the neuromuscular deficits that may arise from concussions and sub-concussive impacts [10,11]. These programs should prioritize balance and proprioception training, incorporating exercises such as single-leg balance drills, perturbation training, and dynamic stability exercises to enhance sensorimotor control and reduce injury susceptibility [9]. Additionally, plyometric training plays a crucial role in refining movement mechanics, with controlled landing drills, deceleration training, and jump mechanics optimization helping athletes develop safer landing techniques, thereby minimizing excessive knee valgus angles, a well-established risk factor for ACL injuries [8].

Furthermore, strength training should emphasize the development of hamstring, gluteal, and core muscle groups, which are essential for maintaining knee stability and counteracting biomechanical vulnerabilities that are particularly prevalent among female athletes [12,34,50]. Given the known impact of concussions on neuromuscular control, cognitive-motor integration training should also be incorporated, utilizing dual-task exercises that combine cognitive challenges with motor tasks to improve reaction time and neuromuscular responsiveness [7]. This approach is critical in mitigating post-concussion impairments that could increase ACL injury risk, ensuring that athletes regain full neuromuscular function before returning to high-risk activities [13,14,15]. By integrating these evidence-based neuromuscular training components, sports medicine professionals can enhance injury prevention protocols and promote long-term athlete safety and performance in female soccer players.

### 4.2. Enhancing Cervical Spine Strength and Stability

Given that female athletes have lower neck strength and smaller head mass compared to their male counterparts, they experience greater head acceleration during impacts, which increases their susceptibility to concussions [5,50]. Strengthening the cervical musculature is therefore essential in dissipating impact forces and reducing the severity of head trauma. Isometric and dynamic neck resistance training using resistance bands and manual resistance techniques can enhance neck muscle endurance and stiffness, which helps in stabilizing the head upon impact [10]. Additionally, postural stability exercises that reinforce head-neck control during dynamic movements, such as perturbation training and controlled head positioning drills, are crucial in improving neuromuscular responses to sudden impacts [8,9].

Furthermore, upper body strength training should be incorporated to enhance overall stabilization during aerial duels and heading. Strengthening the shoulder, upper back, and core muscles contributes to better control of the head-neck complex, reducing the magnitude of rotational and linear accelerations transmitted to the brain upon ball contact [6,8]. Implementing these targeted training strategies within soccer conditioning programs can play a significant role in reducing concussion severity and improving player safety, particularly in female athletes who are at heightened risk due to their biomechanical and physiological differences.

### 4.3. Revisiting Heading Exposure and Technique

To help minimize the cumulative effects of sub-concussive impacts in soccer, specific preventive measures may be beneficial, particularly for female athletes who are at greater risk of concussions and ACL injuries due to biomechanical and neuromuscular differences [17]. One essential step is the implementation of age-based heading restrictions, reducing heading exposure in younger athletes, particularly those under 14, to allow for proper neurodevelopment before introducing repetitive head impacts. Research suggests that delaying exposure to heading in youth soccer can help mitigate the long-term neurological consequences associated with cumulative sub-concussive trauma [6,8].

Another crucial component of injury prevention is heading technique optimization, where coaches should ensure that players are properly trained to engage their neck and core muscles to stabilize impact forces effectively [11]. Studies indicate that players who fail to activate their neck musculature appropriately during heading experience greater head acceleration and force transmission to the brain, increasing concussion risk [5,13]. Additionally, limiting high-risk heading scenarios in training sessions is critical, with an emphasis on controlled heading drills rather than repeated high-impact heading in competitive or fatigued conditions [9,10]. By implementing these targeted interventions, sports medicine professionals and coaches can help mitigate the risks associated with repetitive heading and reduce the likelihood of long-term neurophysiological impairments in female soccer players.

### 4.4. Refining Return-to-Play Protocols

Given the potential for prolonged neuromuscular deficits following concussions, careful structuring of return-to-play protocols could help minimize the risk of subsequent injuries, particularly ACL ruptures [9,16]. One essential component of these protocols is neurocognitive testing, which should include assessments of reaction time, memory recall, and balance stability to determine whether an athlete has achieved full neurological recovery before being cleared to return to play [11,16]. Research has shown that athletes who return to play prematurely, before their neurocognitive function has been fully restored, may exhibit delayed motor responses and impaired coordination, increasing their vulnerability to lower extremity injuries [8,13].

In addition to cognitive assessments, dynamic movement evaluations should be conducted to screen for abnormal biomechanics during cutting, pivoting, and landing, ensuring that players can safely reintegrate into high-intensity gameplay without an elevated risk of ACL injury [5,10]. Athletes recovering from concussions often exhibit altered neuromuscular control and proprioception, which may predispose them to improper knee positioning and instability during these dynamic movements [9]. Furthermore, gradual reintroduction of heading drills should be implemented under controlled conditions, allowing players to progressively regain proper heading technique and neuromuscular coordination before engaging in unrestricted competitive play [14,15]. By incorporating these evidence-based return-to-play strategies, sports medicine professionals can ensure that concussed athletes regain full functional capacity, thereby reducing the likelihood of reinjury and promoting long-term athletic health.

### 4.5. Improving Injury Surveillance and Long-Term Research

To further refine injury prevention strategies and gain a deeper understanding of the long-term impact of concussions on ACL injury risk, comprehensive injury tracking and longitudinal studies are essential. One critical aspect of this approach is enhanced data collection, which can be achieved through the implementation of motion analysis, wearable sensor technologies, and video-based injury surveillance in both training and match settings [18]. These technologies allow for precise measurement of head acceleration, biomechanical changes, and neuromuscular adaptations over time, enabling early detection of movement abnormalities that may contribute to increased injury risk [11,13].

Additionally, longitudinal cohort studies are crucial for tracking athletes over multiple seasons to assess the cumulative effects of concussion exposure on neuromuscular function and ACL injury risk [6,16]. Many current studies only examine short-term outcomes, limiting our ability to understand how repeated head trauma influences neuromuscular control over an athlete’s career. By following athletes over extended periods, researchers can identify trends in injury recurrence, evaluate the effectiveness of intervention programs, and determine the long-term consequences of sub-concussive impacts on movement mechanics [8].

Furthermore, personalized risk assessments should be developed to create individualized injury prevention programs tailored to each athlete’s concussion history, biomechanics, and neuromuscular function [18]. Given the variability in concussion recovery and neuromuscular impairment among athletes, a one-size-fits-all approach is insufficient. Instead, targeted interventions should be designed based on an athlete’s specific risk profile, incorporating neuromuscular training, cognitive-motor integration, and biomechanical corrections to mitigate injury susceptibility [9]. By integrating these advanced research methodologies, sports medicine professionals can develop more effective, evidence-based prevention strategies, ultimately reducing the incidence of both concussions and ACL injuries in female soccer players.

## 5. Limits

While this narrative review aims to provide a comprehensive synthesis of the current evidence on the interplay between concussions, neuromuscular impairments, and ACL injury risk in female soccer players, several limitations must be acknowledged to contextualize its findings and guide future research.

First, the narrative nature of the review inherently limits methodological rigor compared to systematic reviews or meta-analyses. Without a formal protocol such as PRISMA, this review is subject to potential selection bias, as the inclusion of studies was based on thematic relevance and author judgment rather than predefined eligibility criteria and quality appraisal tools. Although efforts were made to include high-impact and recent studies, the absence of a formal grading system may affect the overall strength of evidence.

Second, the search strategy was limited to three major databases (PubMed, Scopus, and Web of Science) and to publications in English, which may have excluded relevant studies published in other languages or indexed in different databases. This introduces a language and publication bias, especially for studies originating from non-English speaking countries with emerging research on female athletes.

Third, while the review emphasizes female soccer players, much of the available literature in this field is still based on mixed-gender cohorts or male-dominant samples, particularly in concussion research. In cases where sex-specific data were not available, the inclusion of broader populations was necessary, which could limit the external validity of the conclusions drawn for female athletes.

Additionally, there is considerable heterogeneity in study designs, outcome measures, and diagnostic criteria among the included articles. Variations in how concussions, neuromuscular deficits, and ACL injuries are defined, measured, and reported make it challenging to compare findings across studies or establish causality. The lack of standardized neuromuscular assessments or consistent use of return-to-play protocols further complicates the interpretation of post-concussive injury risk.

Another limitation is the cross-sectional or retrospective nature of many studies included in this review. Very few longitudinal or prospective designs exist that directly assess the cumulative effects of sub-concussive trauma on lower-extremity injury risk over time. Therefore, while the proposed mechanisms and associations are supported by current evidence, causal inferences must be made with caution.

Lastly, this review did not include a quantitative synthesis of data. The absence of pooled effect sizes or statistical meta-analysis means that relative risk estimates could not be derived. Thus, while the narrative format enables a broad exploration of concepts, it lacks the statistical power to definitively confirm the magnitude of the relationships discussed.

These limitations highlight the need for future research that utilizes standardized methodologies, longitudinal designs, sex-specific sampling, and multimodal assessment protocols. Such approaches will be essential to clarify the neurophysiological mechanisms underlying concussion-related ACL injury risk and to inform more effective prevention and rehabilitation strategies tailored to female athletes.

## 6. Conclusions

The relationship between concussions, sub-concussive impacts from heading, and ACL injuries in female soccer players represents an emerging topic of interest in sports medicine. Repetitive head impacts may be associated with neuromuscular deficits affecting proprioception, reaction time, and postural control, which could increase ACL injury risk. Female athletes, due to physiological and biomechanical differences, are particularly vulnerable. Integrating neuromuscular training, cervical spine strengthening, optimized heading techniques, and improved return-to-play protocols could be beneficial for injury prevention. Longitudinal research and injury surveillance are needed to understand the long-term impact of repetitive heading. Collaboration among sports medicine professionals, coaches, and policymakers is essential to develop evidence-based interventions. A proactive approach will enhance athlete safety, performance, and career longevity. Prioritizing these strategies may support sustained participation in soccer while helping to minimize the risk of injuries.
